# Characterization of the complete chloroplast genome of *Begonia fimbristipula* (Begoniaceae)

**DOI:** 10.1080/23802359.2020.1715872

**Published:** 2020-01-22

**Authors:** Liping Huang, Jinmin Wang

**Affiliations:** a Qinghai University, Xining, Qinghai, China;; b Qinghai Academy of Agriculture and Forestry, Xining, Qinghai, China

**Keywords:** *Begonia fimbristipula*, Begoniaceae, phylogenetic relationship, chloroplast genome

## Abstract

*Begonia fimbristipula* is a plant of perennial flowering plants in the family Begoniaceae. In this article, the complete chloroplast genome of *B. fimbristipula* was studied and illustrated to add more genetic information. The chloroplast genome is 169,436 bp in length as the circular, which exhibits a large single-copy region (LSC) of 77,058 bp, a small single-copy region (SSC) of 18,128 bp, and a pair of inverted repeat regions (IRs) of 37,123 bp in each. The overall nucleotide composition of chloroplast genome is: 31.8% A, 32.6% T, 18.0% C, 17.5% G, and the total GC content 35.5%. A total of 142 genes were annotated that included 91 protein-coding genes (PCGs), 43 transfer RNA (tRNAs), and 8 ribosome RNA (rRNAs). Phylogenetic relationship shows that *Begonia fimbristipula* is more closely related to *Begonia pulchrifolia* on genetic relationships using the Maximum-Likelihood (ML) method.

Begonia is one of the most diverse plant taxa in the world, which consists of over 1800 accepted species. The number of species recognized within the genus Begonia has greatly increased over the past 20 years, rising from 80 to 200 species only in China (Tian et al. 2018). *Begonia fimbristipula* (Begoniaceae) is a stemless perennial herb, which is mainly distributed in Southern China. *Begonia fimbristipula* is cultivated as a popular commercial herbal tea in Guangdong Province (Tebbitt 2005). The leaves, dried stem, and flower of *Begonia fimbristipula* are used in Chinese herb to reduce inflammation, eliminate phlegm, and relieve cough and asthma (Han et al. 2013). However, little is known about the genome information of *B. fimbristipula* that can provide more data available to study this species. So, this article has finished the chloroplast genome of *B. fimbristipula*, which can be useful for genetic evolution and utilization of germplasm resources of the family Begoniaceae in the future.

The specimen fresh leaves sample of *Begonia fimbristipula* was collected from the market of Jiufeng town, Guangan city, Sichuan Province, China (106.88E; 30.13 N). Total genomic DNA of *B. fimbristipula* was extracted from the fresh leaves tissue using Plant Tissues Genomic DNA Extraction Kit (TIANGEN, BJ and CN) and stored in the Horticultural Institute of Qinghai Academy of Agriculture and Forestry (No. HI-QH-AAF-01). The chloroplast DNA of *B. fimbristipula* was purified and sequenced by the sequencer that the collected raw sequences were quality controlled and removed by the FastQC (Andrews 2015). The chloroplast genome of *B. fimbristipula* was assembled and annotated by the MitoZ (Meng et al. 2019). The chloroplast genome map was generated by the OrganellarGenomeDRAW (Lohse et al. 2013).

The complete chloroplast genome of *Begonia fimbristipula* was from the GenBank with the accession No. MN076318 as reference genome sequence, which is a 169,436 base pair (bp) in length as a circular that has the characteristic quadripartite structure. It consists of a large single-copy region (LSC of 77,058 bp), a small single-copy region (SSC of 18,128 bp) and a pair of inverted repeat regions (IRs of 37,125 bp in each). The overall nucleotide composition of the chloroplast genome is 31.8% of A, 32.6% of T, 18.0% of C, 17.5% of G, and the total GC content of 35.5%. The chloroplast genome of *B. fimbristipula* contains 142 genes, which includes 91 protein-coding genes (PCG), 43 transfer RNA genes (tRNAs), and 8 ribosomal RNA genes (rRNAs). Each 28 genes were found duplicated in IR regions, which includes 12 PCGs species (*psbI, psbK, matK, psbA, rpl2, rpl23, ycf2, ndhB, rps7, rps12, ycf15, a*nd *ycf1*), 12 tRNAs species (*trnG-UCC, trnS-GCU, trnQ-UUG, trnK-UUU, trnH-GUG, trnI-CAU, trnL-CAA, trnV-GAC, trnI-GAU, trnA-UGC, trnR-ACG,* and *trnN-GUU*), and 4 rRNAs species (*rRNA16, rRNA23, rRNA4.5,* and *rRNA5*).

To analyze the phylogenetic relationship of the family Begoniaceae, 15 species chloroplast genomes were used to construct the phylogenetic tree by the Maximum-Likelihood (ML) method. ML analysis of the phylogenetic tree was performed using the MEGA X software (Kumar et al. 2018) with GTR + G+I model and all of the nodes were inferred with strong support by 2000 bootstrap values replicate for each node. The phylogenetic tree was represented using the MEGA and edited using the Evolview online (www.evolgenius.info/evolview) (Subramanian et al. 2019). Phylogenetic relationship shows that *Begonia fimbristipula* is more closely related to *Begonia pulchrifolia* on the genetic relationship (MN076318) in the evolutionary relationship (Figure 1). This paper offers the importance of the medicinal valuable study and chloroplast genome information of the family Begoniaceae.

**Figure 1.**
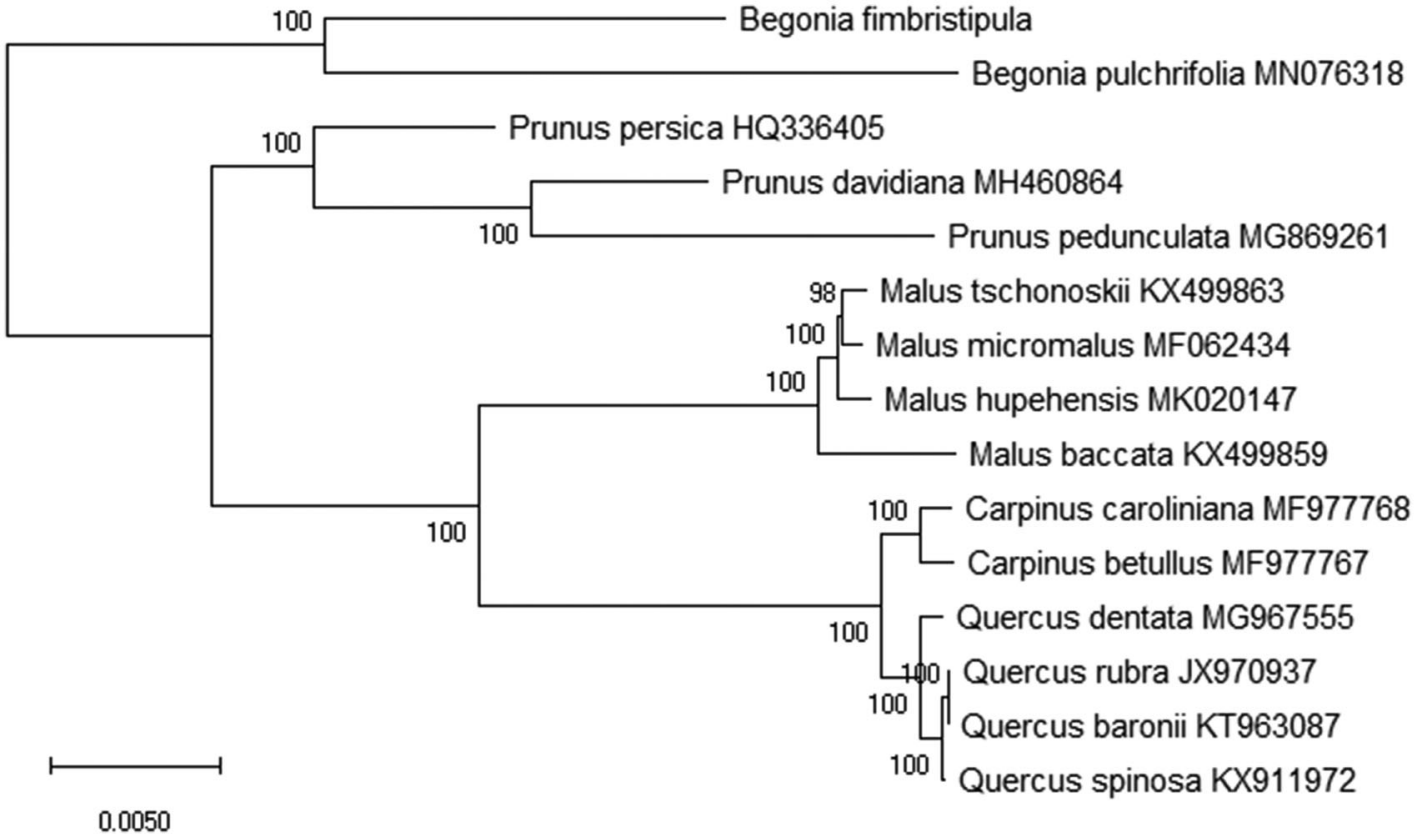
The maximum likelihood tree of *Begonia fimbristipula* based on 15 species chloroplast genomes. All the nodes are the bootstrap values from 2000 replicates.
